# Rib soft fixation produces better analgesic effects and is associated with cytokine changes within the spinal cord in a rat rib fracture model

**DOI:** 10.1177/1744806919855204

**Published:** 2019-06-04

**Authors:** Yuan-Yuarn Liu, Jeffrey Chi-Fei Wang, Ya-Chi Lin, Hung-Tsung Hsiao, Yen-Chin Liu

**Affiliations:** 1 Division of Trauma, Department of Emergency, Kaohsiung Veterans General Hospital, Kaohsiung City; 2 Department of Anesthesiology, National Cheng Kung University Hospital, College of Medicine, National Cheng Kung University, Tainan City

**Keywords:** Rib fracture, analgesia, cytokine, rat, glia

## Abstract

Traumatic rib fracture can cause severe pain and is usually associated with the depression of respiratory drive followed by severe respiratory complications. It is critical for patients with rib fracture to receive adequate analgesia. However, strong opioids and other analgesics often produces side effects and may even cause respiratory suppression. Meanwhile, rib fixation now has become a popular method for treating rib fracture patients. However, the actual molecular mechanism leading to its effectiveness as an analgesia has not been fully investigated, and the best analgesic method for its use in rib fracture patients has not yet been determined. We developed a new animal model for rib fracture and evaluated changes in pain severity after rib fixation. Our data indicated significantly better analgesic behavior if a soft string rib fixation is performed, which is associated with cytokine (interleukine-6 and interleukine-10) decreases in the spinal cord and co-localization with glia cells. Our results provided a treatment suggestion for rib fracture patients and the possible molecular mechanism for the analgesic effects. Further molecular mechanisms and the best therapeutic methods are still needed for this severe painful condition.

## Background

Chest wall trauma is relatively common and contributes significantly to morbidity and mortality in trauma patients. If multiple rib fractures occur with flail chest for such patients, which is generally defined as three or more ribs fractured, this is associated with a higher risk of mortality.^1^ Usually, the number of displaced rib fractures could be a strong predictor for developing pulmonary complications.^2^ Even minor rib fractures (less than two ribs fractured) make patients susceptible to pneumonia, particularly in the case of elderly patients and those with chronic obstructive pulmonary disease.^3^ However, even if various methods to repair rib fractures are proposed,^4^ treatment for severe chest wall injury remains mainly nonsurgical and most of such treatments lack adequate analgesia.^1^ Treatment for flail chest injury including epidural catheter analgesia comprises 8% of such patients, and surgical fixation for chest rib fracture comprises only 0.7%.^1^ Meanwhile, rib fixation has been proved to shorten the length of stay in hospital and is associated with improved lung function.^5,6^ However, the best analgesic method has yet to be investigated thoroughly, and the molecular mechanism is also lacking because of a lack of an available animal model. Thoracic epidural, thoracic paravertebral, and intercostal blocks are also suggested for patients who received regional blocks.^7^ Parenteral or oral opioids are also a simple choice but may be associated a higher pain score or the immune response.^8,9^ Therefore, it is important for clinicians to investigate the best analgesic method for rib fracture patients. We created a new animal model for rib fracture to evaluate pain behavior and the analgesic mechanism, where it is hypothesized that non-rigid soft string fixation of the rib can improve pain severity in rib fracture animals and that this improvement in the analgesic effect is associated with cytokine changes in the spinal cord.

## Methods

### Rat rib fracture and soft fixation model

Sprague Dawley (SD) rats obtained from National Cheng Kung Medical College Animal Center or BioLASCO Taiwan Co. were used in this study. All procedures were approved according to the National Cheng Kung Medical College Animal Care Guidelines (IACUC Approval No: 106195). The model was modified from our previous chronic post-thoracotomy pain model.^10^ Rats were in left lateral position under light isoflurane anesthesia (1%–2%) with 100% mask oxygen supply. A skin incision of approximately 2 cm was created along the rib just on the middle axillary line. The third or fourth rib was identified with the landmark just under the scapular. A sharp scissor was used to cut through the third or fourth rib to make the rib move free. The rib fixation group was fixed via 4–0 nylon string with cutting needle puncture through the rib. The rib could still move but movement was limited (soft fixation). Then, the skin was closed with 4–0 nylon string and removed three days later to avoid stitch-induced discomfort. After recovery from surgery, rats were closely observed and their bodyweight change and further pain behavior testing were checked. No analgesic drug was given for all animals. All animals were also checked via X-ray on the seventh day to confirm the right rib fracture site. Any rib fractures that were not in third or fourth rib were excluded.

### Fracture pain behavior testing

The pain behavior measurements for rib fracture were similar to those in our previous post-thoracotomy research.^11^ Each rat was placed in a loose restraining cage (8 cm × 9 cm × 20 cm) to test mechanical hyperalgesia and allowed to rest there for 15 min. A series of calibrated von Frey filaments (VFH; Stoelting Co, Wood Dale, IL) with bending forces ranging from 0.4 to 15.0 g was applied perpendicularly to the dorsal skin surface, starting from the lowest force, to calculate the threshold for a nocifensive response (slight movement of feet to shift body away from the stimulus or local subcutaneous muscle contraction; strong trunk muscle response and brisk lateral movement of the trunk away from the stimulus; scratching near the test location, whole body shuddering or vocalization).^11^ Each VFH was applied sequentially around the fracture region. Each testing spot, separated by 1cm, was probed twice, pressing with a 3-s duration spaced 3-s apart. There was a 30-s resting period between each spot tested. The VFH force was increased progressively until a defined response occurred and then reduced and again increased to verify the threshold force. A response to the lowest force filament that was used, 0.4 g was assigned as the threshold, and those not responding to the highest force were assigned a “ceiling” threshold of 16 g. Higher forces were avoided to minimize the tactile sensitization that occurs with these stiffer VFHs. The four quarters around the bone-fracture skin incision site was checked and recorded.

### Bone healing evaluation

The bone evaluation was performed via a high-resolution X-ray micro-CT system (SKYSCAN 1076, Belgium). Each rat was followed up with an X-ray on the 7th, 28th, and 56th day to evaluate serial changes in rib healing. Microtomography (micro-CT) was also used to evaluate the actual bone mineral density (BMD) at the end of the experiment (56th day).

### Protein expression on spinal cord

The dorsal horn of spinal cord (T3–T4 area) was harvested on day 16 and day 56 to evaluate the cytokine and cell expression over the sensory area of the ipsilateral spinal cord via Western blotting methods according to our previous methods.^12,13^ Briefly, the animals were rapidly euthanized after deep anesthesia, and the spinal cord areas (T3–T4) were quickly removed. The tissues were sonicated and incubated on ice after the addition of a lysis buffer, a protease inhibitor cocktail, and a phosphatase inhibitor cocktail. Following centrifugation, the supernatants were collected and stored at −80°C. Then, the proteins were subjected to sodium dodecyl sulfate-polyacrylamide gel electrophoresis with precast 4% to 12% mini gels with the NuPAGE Bis-Tris buffer system (Invitrogen, Carlsbad, CA). After electrophoretic separation, the proteins were transferred to polyvinyl denefluoride membranes (Millipore, Bedford, MA) and then incubated overnight at 4°C with primary antibodies against glial fibrillary acidic protein (GFAP, marker of astrocytes, 1:10,000, rabbit; Millipore), Iba-1 (marker of microglia, 1:1000, rabbit; Wako, Richmond, VA), tumor necrosis factor-α (TNF-α; 1:1000, rabbit; GeneTex), interleukin-1β (IL-1β; 1:1000, rabbit; Abcam), interleukin-6 (IL-6; 1:1000, rabbit; Abcam), interleukin IL-10 (IL-10; 1:1000, rabbit; Abcam), and actin (1:10,000, mouse; Millipore). After the primary antibody incubation, the blots were washed with 0.05% PBS-Tween 20 and detected with the respective secondary antibody, either anti-mouse or anti-rabbit IgG (GE Healthcare Life Sciences, Little Chalfont, UK) linked to horseradish peroxidase at room temperature for 60 min. Chemiluminescence detection was performed with Immobilon Western Chemiluminescent HRP Substrate (Millipore Corporation) and measured directly using a BioSpectrum Imaging System (UVP, Cambridge, UK).^13^

### Immunofluorescence stained for cytokine cell co-localization

The animals were anesthetized prior to transcardial perfusion with 4% paraformaldehyde. The spinal cord area of the fractured rib (T3 or T4) was removed, immersed in 4% paraformaldehyde for 2–4 h, and then placed in 20% sucrose for 48 h at 4°C. The spinal cord sections were cut to 10 μm and attached on slides for immunofluorescence. The sections were blocked with antibody diluent with background reducing components (Dako) for 1 h at room temperature and then stored overnight at 4°C with primary antibodies against Iba-1 (mouse, 1:850, Abcam), IL-6 (1:650, rabbit, GeneTex), IL-10 (1:850, rabbit, Abcam), and the cell nucleus (Prolong™ Gold antifade with DAPI, Invitrogen). After incubation in the primary antibody, the sections were washed three times and incubated with appropriate fluorescent secondary antibodies (1:2000, AlexaFluor 488, 594, Invitrogen) for 2 h at room temperature. The stained sections were then examined using fluorescence microscopy.

### Quantification and statistics

For the behavioral studies, the data for the withdrawal thresholds passed a normality test, and thus were deemed suitable for parametric statistics. The data were analyzed with a Student’s t-test (two groups only). All data were presented as mean ± standard error of the mean (SEM), and *P* < 0.05 was considered statistically significant. For the quantification of the Western blot, the density of specific bands was measured with imaging analysis software (Image J, NIH). The size of the rectangle was fixed for each band, and the background near the band was removed. The expression level of each protein was normalized to loading controls (β-actin). The Western blot data were also analyzed with a Student’s t-test (two groups only).

## Results

### Soft rib fixation provided better analgesia compared with non-fix group

**Figure 1.**
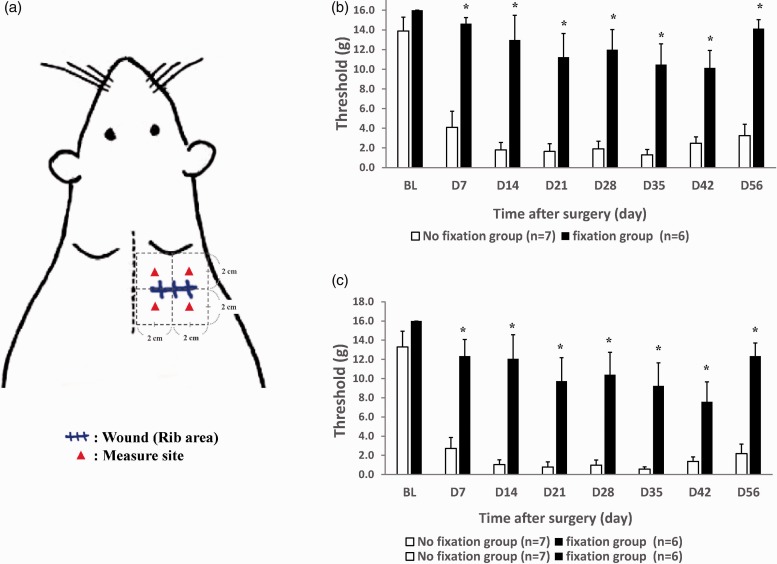
Rats with soft fixation over the fracture rib produces less pain behavior compared with non-fix rib fracture rats. (a) The measure site for animal. (b) (Mean) and (c) (Least) Mechanical hyperalgesia behavior response around the rib fracture site after rib fracture to 56 days. Student’s t-test, *means *P* < 0.05, compared to non-fix group, n = 6–7 rats. Data were presented with mean ± SEM.

The comparison withdrawal threshold for the two different groups of rib fracture animals is presented in Figure 1. The detailed measure site for animals was presented in Figure 1(a). We measured the average (Figure 1(b)) and minimum (Figure 1(a)) pain withdrawal threshold over the four quarters around the fracture site. The string fix rib fracture animals exhibited better analgesic responses compared with the non-fix group after the initial week. On day 7, the average mechanical pain withdrawal threshold (Figure 1(a)) was 14.6 ± .62 (fix) versus 4.1 ± 1.64 (non-fix) (*P* < 0.01), which gradually decreased to 10.1 ± 1.77 versus 2.5 ± .65 (*P* < 0.01) on day 42. This demonstrated strong evidence of the benefits of rib fixation.

### Osteogenic density checked by X-ray presented no difference for rib fracture animals with or without fixation

**Figure 2.**
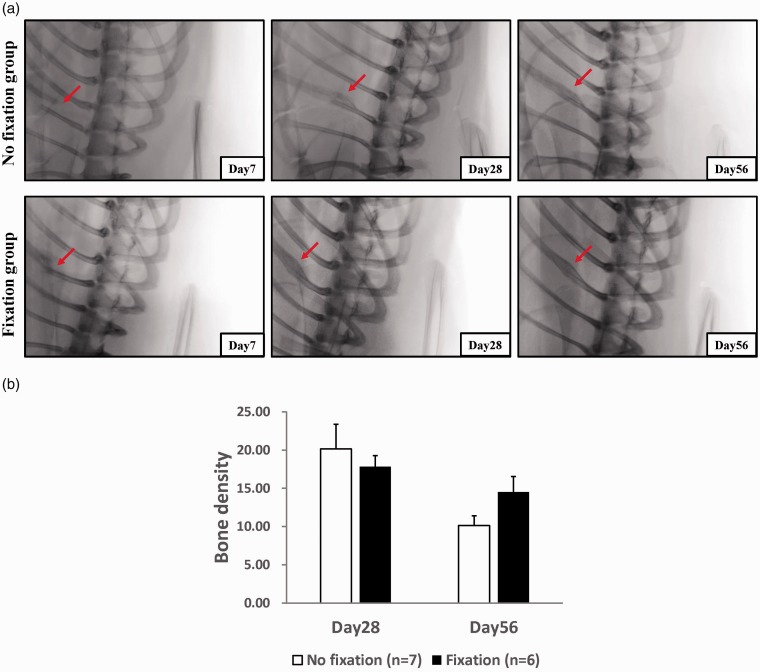
X-ray check for rib fracture and the densitometry analysis. Day 7 was used to confirm the fracture location (third or fourth rib) and day 28 and day 56 X-ray check after rib fracture with or without fixation (a) and densitometry (b) check after subtraction background. Student’s *t*-test without statistical significance between groups, n = 6–7 rats. Data were presented with mean ± SEM.

As a clinical situation, we checked the osteogenic recovery of the rib fracture for pain sensation via X-ray to demonstrate possible recovery. However, our data showed no between-group differences in rib formation density (Figure 2). On day 28, the bone density (after background subtraction) was 17.83 ± 1.42 (fix group) versus 20.14 ± 3.21 (non-fix group) (*P* = 0.55) and on day 56, it was 14.50 ± 2.05 (fix group) versus 10.14 ± 1.26 (non-fix group) (*P* = 0.09). This also demonstrated that an X-ray examination is not a suitable tool for rib fracture for the purpose of pain evaluation.

### Soft rib fixation provided better bone density formation according to the Micro-CT evaluation

**Figure 3.**
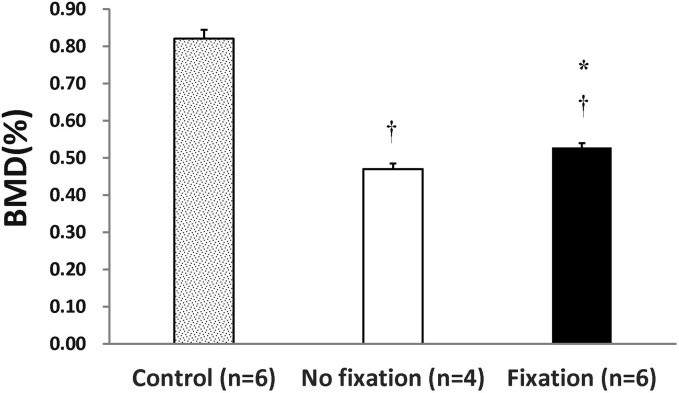
Micro-CT demonstrated the soft fix rib produce better bone density than non-fix group. BMD checked by micro-CT between two groups of rats with rib fracture on day 56. All bone density of fracture rib is less than the non-fracture rib and the soft fix rib produce better bone density than non-fix rib. Student’s *t-*test, ^†^means *P* < 0.05, compared to control group, *means *P* < 0.05, compared to non-fix group, n = 4–6. Data were presented with mean ± SEM. BMD: bone mineral density.

Although the X-ray examinations showed no between-group differences, the Micro-CT indicated better BMD formation on day 56 in the soft fix group 0.53 ± .012 (fix group) versus 0.47 ± .015 (non-fix group) (*P* = 0.016). However, both groups still exhibited less density than the control (no fractured bone) (0.82 ± .024) (Figure 3).

### On both day 16 and day 56, cytokine IL-6 and IL-10 in the spinal cord samples were less expressed in the fix group, but the effects were not obvious in the TNF-α, IL-1β and cell markers

**Figure 4.**
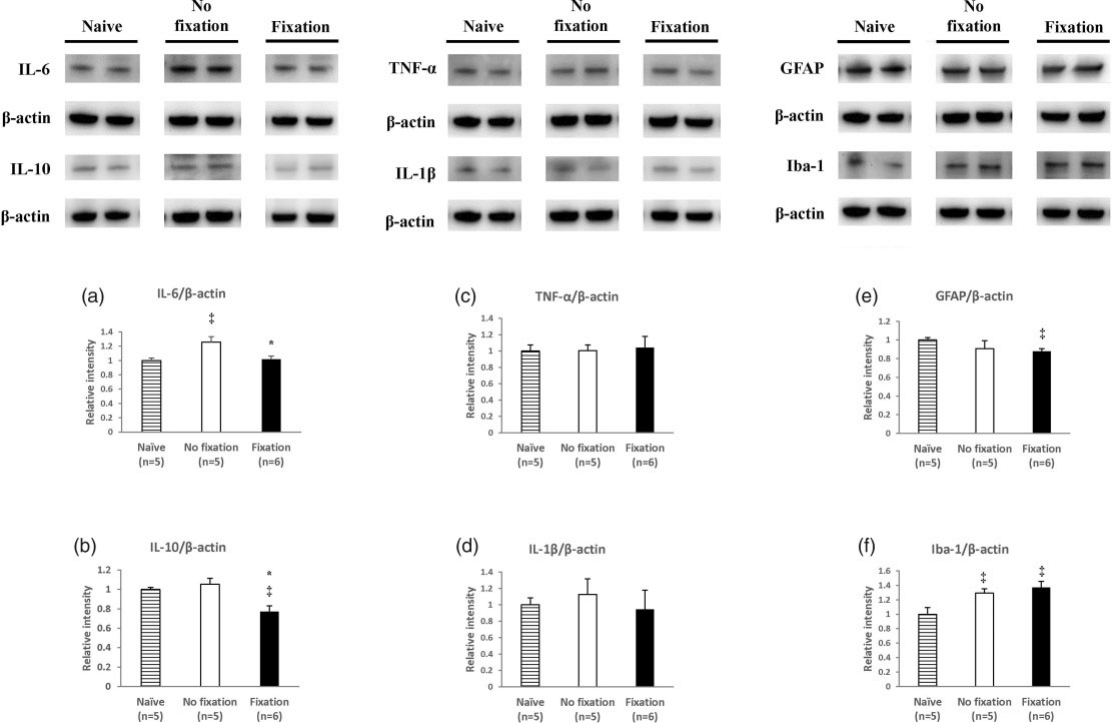
Pro-inflammatory cytokine changes between soft fix and non-fix groups in spinal cord on day 16. Western blotting analysis showing IL-6, IL-10, GFAP, Iba-1, TNF-α, and IL-1β expression in spinal cord dorsal horn on day 16. Student’s *t*-test, ^‡^means *P* < 0.05, compared to Naïve group, *means *P* < 0.05, compared to non-fix group, n = 5–6 mice. Data were presented with mean ± SEM. GFAP: glial fibrillary acidic protein; Iba-1: ionized calcium binding adaptor molecule 1; TNF-α: tumor necrosis factor-α; IL-1β: interleukin-1β; IL-6: interleukin-6; IL-10: interleukin.

**Figure 5.**
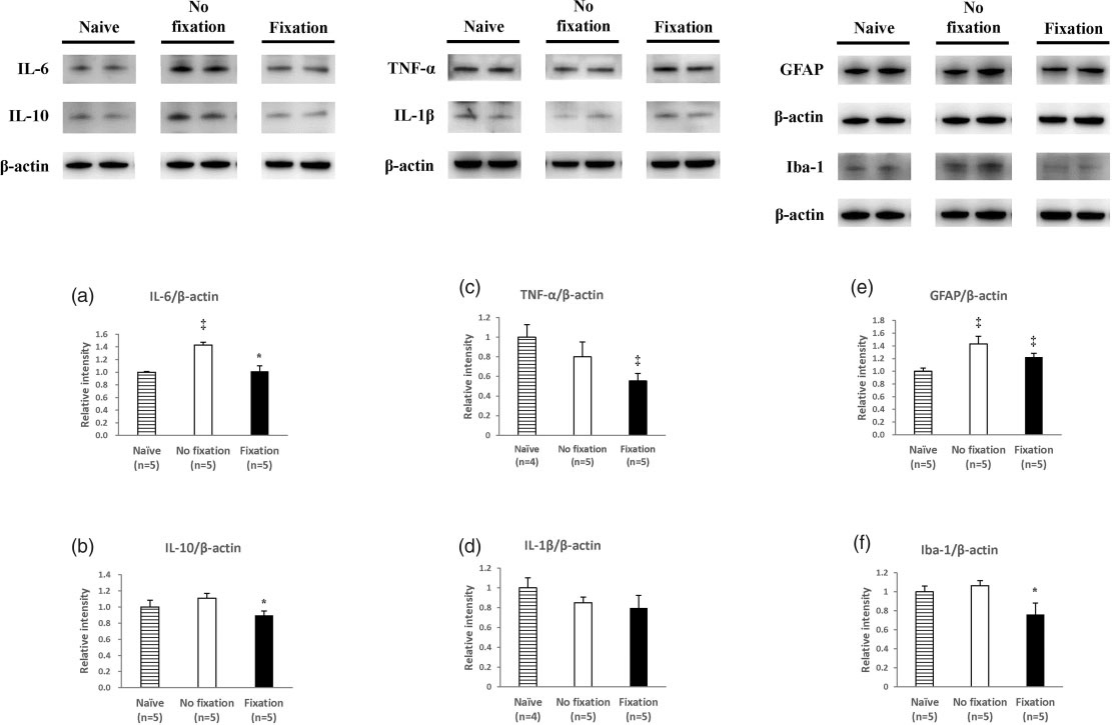
Pro-inflammatory cytokine changes between soft fix and non-fix groups in spinal cord on day 56. Western blotting analysis showing IL-6, IL-10, GFAP, Iba-1, TNF-α, and IL-1β expression in spinal cord dorsal horn on day 56. Student’s *t*-test, ^‡^means *P* < 0.05, compared to Naïve group, *means *P* < 0.05, compared to non-fix group, n = 5–6 mice. Data were presented with mean ± SEM. GFAP: glial fibrillary acidic protein; Iba-1: ionized calcium binding adaptor molecule 1; TNF-α: tumor necrosis factor-α; IL-1β: interleukin-1β; IL-6: interleukin-6; IL-10: interleukin.

On day 16, there was a difference in the levels of IL-6 and IL-10 between the fix and non-fix groups (IL-6: (Naive: 1.00 ± .035), (non-fix: 1.26 ± .075, fix: 1.02±.045, *P =* 0.02)) and (IL-10: (Naive: 1.00 ± .022), (non-fix: 1.06 ± .063, fix: 0.77 ± .058, *P =* 0.01)). However, there was no between-group difference in the TNF-α, IL-1β, and cell markers (GFAP and Iba-1) (Figure 4). On day 56, compared with the non-fix group, the fix group still had less pro-inflammatory cytokine IL-6 and IL-10 in the spinal cord dorsal horn (IL-6: (Naive: 1.00±.011), (non-fix: 1.43±.047, fix: 1.01± .086, *P <* 0.01)) and (IL-10: (Naive: 1.00±.084), (non-fix: 1.11± .058, fix: 0.90 ± .055, *P =* 0.03)). Differences were also found in the glia cell marker (Iba-1): (Iba-1, (Naïve: 1.00 ± .059), (non-fix: 1.06 ± .052, fix: 0.76 ± .121, *P* = 0.04)). However, there were no between-group differences in the TNF-α, IL-1β, and astrocyte cell markers (GFAP) (Figure 5).

### Cytokine IL-6 and IL-10 exhibited co-localization with glia (Iba-1) in the spinal cord after the rib fracture but not astrocyte

**Figure 6.**
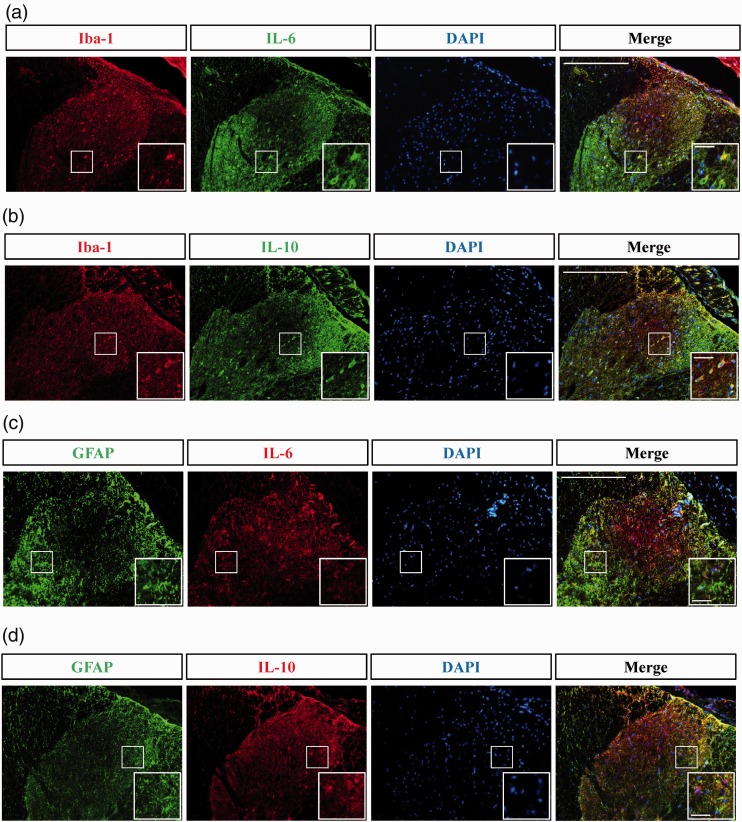
Double immunofluorescence staining for cell and cytokine co-localization after rib fracture. Those data clearly demonstrated that, after rib fracture, IL-6 (a and c) and IL-10 (b and d) presented with microglia cells (Iba-1) but not astrocytes (GFAP). Scale: 200 µm and 25 μm, respectively. Iba-1: ionized calcium binding adaptor molecule 1; IL-10: interleukin; DAPI: 4,6-diamidino-2-phenylindole.

We also used double staining to check co-localization of cytokines with cells after rib fracture and found that both IL-6 and IL-10 were co-localized with glia cells (Figure 6(a) and (b)) but not astrocytes (Figure 6(c) and (d)). It was posited that glia cells but not astrocytes may play a major role in rib fracture-induced pain. The time series staining quantification of IL-6, IL-10, GFAP, and Iba-1in spinal dorsal horn for rib fracture rats were also presented (Supplementary Figure 1).

## Discussion

To the best of the author’s knowledge, this is the first rib-fracture rodent model for pain evaluation. It demonstrated that soft rib fixation provides better analgesic effect as compared to non-fixation and that this analgesic effect is associated with proinflammatory cytokine reduction in the spinal cord. It is well known that a rib fracture is a painful and hard-to-treat, for which conservative oral analgesic therapy^7^ is recommended and which usually requires opioids.^14^ However, recently, patient care has involved a multidisciplinary approach that includes surgical fixation.^15^ We used an easy method (soft fixation) and demonstrated better analgesic effects and a reduction in cytokine within the spinal cord, which suggests a possible new method for treating and understanding this painful condition, which is the novel finding of this research.

Although it was easy to diagnose a rib fracture in our animal model via X-ray, in clinical settings, X-ray have been proven to have a only 37.7% detection rate via the frontal view.^16^ Another study demonstrated that chest radiography found all injuries in only 29.0% of injured patients.^17^ However, early detection and observation are very important in such cases, and chest computed tomography should be arranged if rib fractures are suspected. It is also hinted that rib recovery diagnosed via X-ray is not related with pain sensation after rib fracture. We also now know that congenital anomalies, including supernumerary or accessory ribs, vestigial anterior ribs, bifid ribs, and synostoses, are common and should not be confused with traumatic pathologic conditions. Also, nontraumatic injuries may mimic traumatic rib injuries and may also include metastatic disease, primary osseous neoplasms, fibrous dysplasia, and Paget’s disease.^18^

Our results also demonstrated via micro-CT examination that cytokine changes in IL-6 and IL-10, but not in TNF-α or IL-1ß, in the spinal cord are related to rib fracture pain sensation and that a soft fixation rib exhibits better bone density. One may wonder is such changes are related to bone healing and not pain. However, the anti-IL-6 receptor antibody has been proven to prevent loss of bone structure and bone strength,^19^ and IL-6 knockout mice have demonstrated significantly reduced osteoclastogenesis.^20^ It has also been demonstrated that a fracture can increase IL-10^21^ and contribute to bone healing.^22^ Therefore, it is suggested here that changes in IL-6 and IL-10 in the spinal cord must be related to pain. Certainly, IL-6 is still a potential therapeutic target for pain,^23^ and IL-10 in the spinal cord is also involved in chronic neuropathic pain.^24^ The lower expression marker of glia (Iba-1) on day 56 also suggests that neuroplasticity is involved in rib fracture pain.^25^

In conclusion, we present a novel animal pain model that resembles clinical rib fracture patients. We also demonstrate that soft fixation for a fracture rib produces better analgesic effects and is associated with cytokine changes in the spinal cord. These data provide an important understanding of the pain induced by rib fracture and a possible method by which to treat this clinical situation. Further randomized clinical trials are needed to find the best treatment for this common painful condition.

## Supplemental Material

Supplemental material for Rib soft fixation produces better analgesic effects and is associated with cytokine changes within the spinal cord in a rat rib fracture model Click here for additional data file. Supplemental Material for Rib soft fixation produces better analgesic effects and is associated with cytokine changes within the spinal cord in a rat rib fracture model by Yuan-Yuarn Liu, Jeffrey Chi-Fei Wang, Ya-Chi Lin, Hung-Tsung Hsiao and Yen-Chin Liu in Molecular Pain

## Authors’ Note

YY Liu and JCF Wang provided the idea and performed the lab work; HT Hsiao wrote the article and provided part of the idea; YC Lin performed lab work; and YC Liu contributed details and wrote the manuscript.
