# Supplementary figures and images for: Rib soft fixation produces better analgesic effects and is associated with cytokine changes within the spinal cord in a rat rib fracture model
Source: Mol Pain. 2019 Jun 4;15:1744806919855204. doi: 10.1177/1744806919855204 (PMC6552368; doi:10.1177/1744806919855204)

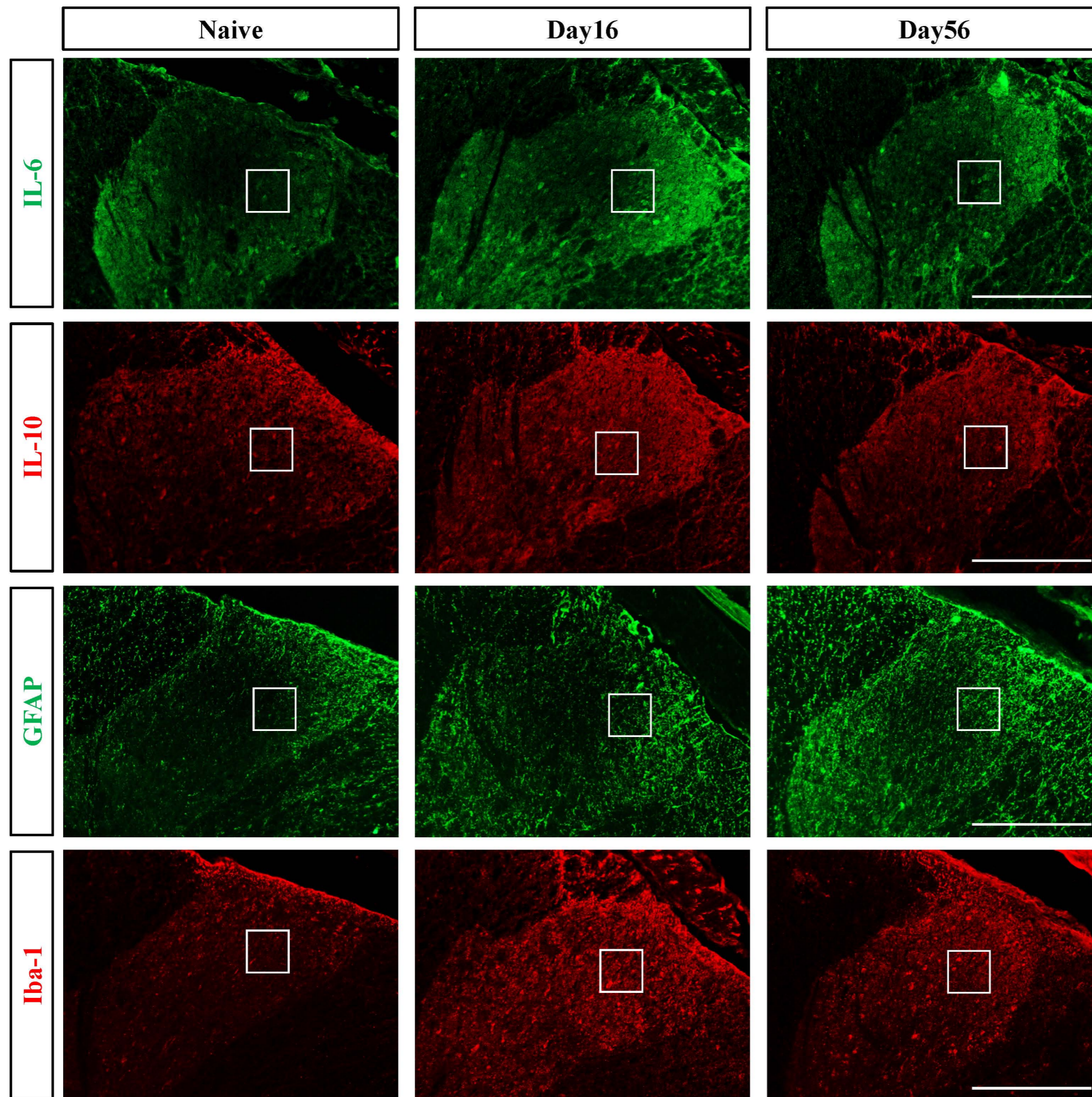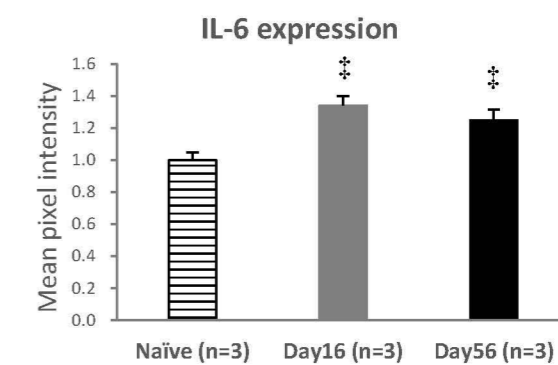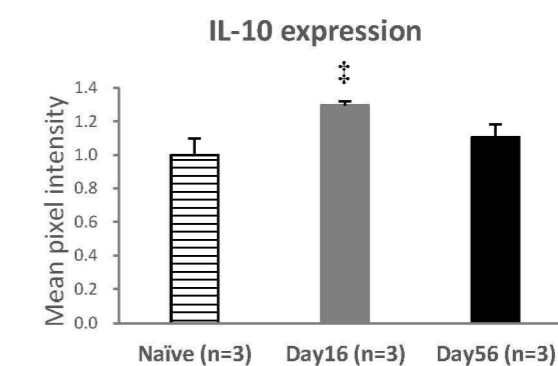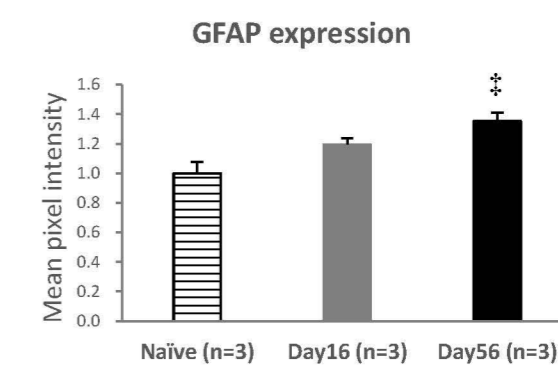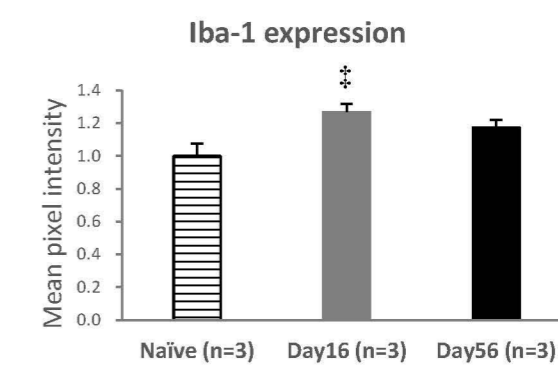

Supplement: Supplemental material for Rib soft fixation produces better analgesic effects and is associated with cytokine changes within the spinal cord in a rat rib fracture model [file Supplemental_Material.pdf]
